# Asymptomatic Fusiform Aortic Aneurysms in a 37-Year-Old Male: A Case Report

**DOI:** 10.7759/cureus.44358

**Published:** 2023-08-30

**Authors:** Rabail Saeed Shaikh, Shujee Yawar, M. Akram Khan

**Affiliations:** 1 Cardiology, Cardiac Center of Texas, PA, McKinney, USA

**Keywords:** case reports, asymptomatic, young male adults, fusiform aneurysms, abdominal aortic aneurysms

## Abstract

Abdominal aortic aneurysm (AAA) is mostly present in patients aged ≥65 years. Here, we present an unusual case of a 37-year-old male with a pair of asymptomatic, fusiform abdominal aortic aneurysms above and below the origin of the renal arteries. The patient was diagnosed with AAA in 2016 and had undergone yearly follow-ups since then. He had no major risk factors for AAA other than hypertension, which was managed with medication, and had only a brief history of smoking. He was also negative for all genetic and connective tissue defects. His aneurysms progressed slowly, with proximal and distal aneurysms currently measuring 3.9 cm and 4.5 cm, respectively. The patient was asymptomatic and was closely examined for further management.

## Introduction

An abdominal aortic aneurysm (AAA) is a permanent, localized aortic dilatation with a diameter greater than 3.0 cm. AAAs measuring 2.9-4.9 cm most commonly range from 1.3% in middle-aged males to 12.5% in geriatric men and from 0% to 5.2% in women [1]. The yearly incidence rate for AAA is 0.89 per 100,000 persons aged 44 and under [2], and the incidence is even lower in Asians. In our case, the asymptomatic presentation of the patient was far from the typical presentation with which patients usually present, commonly with a ruptured aneurysm.

## Case presentation

A 44-year-old male arrived at our clinic for an annual cardiac visit. He had recently relocated to the United States and needed his blood pressure medication to be renewed.

His medical history included hypertension diagnosed in 2014 after which the patient was prescribed telmisartan 40 mg, which he was taking inconsistently. At the age of 37, he was diagnosed with an AAA which was found incidentally upon abdominal computed tomography (CT) scan in 2016 when he was rushed to the hospital due to profuse sweating, high blood pressure, and an overall sense of malaise. The following year in July 2017, a CT scan of the abdomen revealed two closely spaced, non-complicated fusiform aneurysms of the abdominal aorta (figure eight appearance). With a maximum diameter of 3.2 cm, the proximal aneurysm affected the suprarenal aorta shortly after the origin of the superior mesenteric artery, and with a maximum diameter of 3.1 cm, the distal aneurysm affected the infrarenal aorta shortly after the origin of the renal arteries. Both aneurysms were separated by a 2.1 cm section of the normal aorta. Because of his AAA, the patient stated that he had yearly check-ups with his doctor. An abdominal CT performed in August 2021 revealed that both aneurysms had grown, with the proximal aneurysm measuring 3.6 cm and the distal aneurysm measuring 4.2 cm. A partial eccentric thrombosis with a maximum width of 1.5 cm was also discovered in the left lateral wall of the distal aneurysm. The diameter of the aorta between aneurysms also enlarged to 2.6 cm and there was no evidence of narrowing of the renal artery.

On this appointment, he reported no symptoms other than a periodic rise in blood pressure. The patient was also a former smoker who smoked between 1999 and 2000. Except for his blood pressure of 159/90 mmHg and borderline high cholesterol levels, his vital signs, body mass index of 23.5 kg/m^2^, and electrocardiogram were all normal. He was instructed to continue taking telmisartan 40 mg and to maintain a blood pressure journal. An aortic duplex ultrasound was scheduled in two weeks.

**Figure 1:**
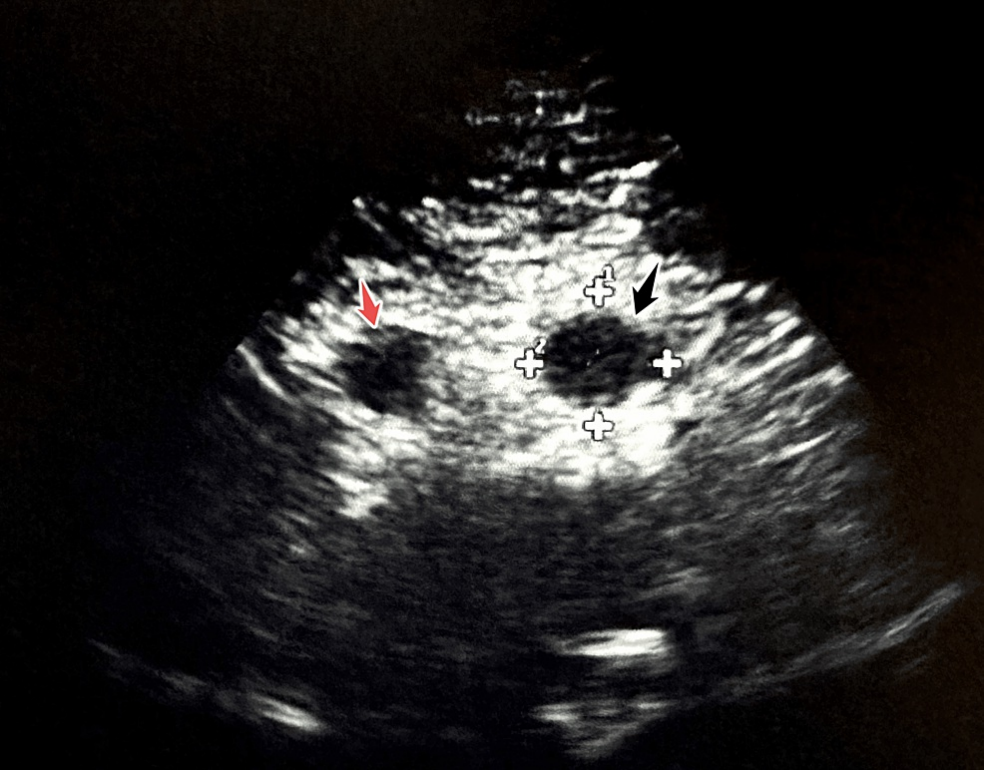
Abdominal ultrasound demonstrating the suprarenal (red arrow) and the infrarenal (black arrow) aneurysm separated by the aorta measuring 2.9 cm.

**Figure 2:**
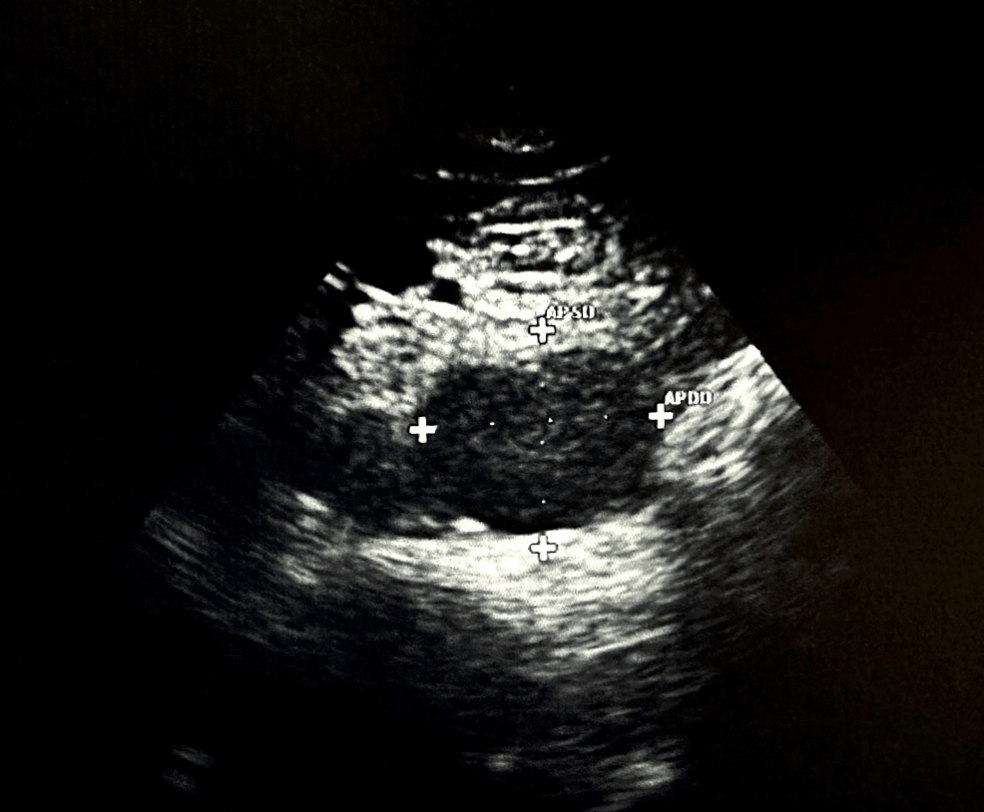
Abdominal ultrasound demonstrating the suprarenal aortic aneurysm measuring 3.8 cm in the proximal aorta.

**Figure 3:**
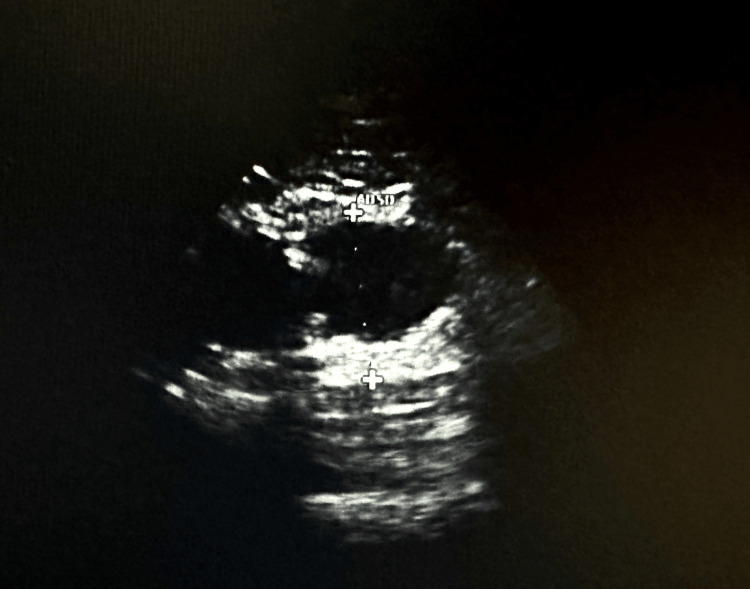
Abdominal ultrasound demonstrating the infrarenal aortic aneurysm measuring 4.4 cm in the distal aorta.

On follow-up ultrasonography, two fusiform aneurysms in the abdominal aorta were discovered (Figure 1). The proximal aneurysm was 3.8 cm wide (Figure 2), while the distal aneurysm was 4.4 cm wide (Figure 3). The aorta measured 2.9 cm. No signs of dissection were noted, and his blood pressure was well-controlled with his current medication. No previous illnesses or serious hospitalizations were reported. A computed tomography angiography (CTA) of the abdomen was scheduled after three months; however, the patient was a no-show and did not return to the clinic until eight months later.

Eight months later, the patient returned to the clinic with no symptoms. He mentioned being tested for genetic diseases in October 2022, which was negative for all connective tissue disorders. This visit revealed that the proximal and distal aneurysms had grown to 3.9 cm and 4.5 cm, respectively. Because of his elevated triglyceride levels of 169 mg/dL, he was also placed on Lipitor 10 mg. He was told to return after three months for a lipid profile, complete metabolic profile, and CTA of the chest/abdomen. The management will be based on the upcoming CTA report.

The treatment strategy was discussed in depth with the patient explaining that if the aneurysms are larger than 5.0 cm or close to 4.5 cm, intervention with endovascular grafting will be performed if they satisfy the criteria.

## Discussion

This case piques our interest because of the patient’s young age and asymptomatic presentation, which differs significantly from the typical presentation of AAA, as it opens possibilities for further research into the countless variables that could have contributed to its pathophysiology and unusual nature. Typically, AAA is discovered late when a patient develops symptoms such as dull pain in the abdomen, flank, or back. Other manifestations may include limb ischemia or an inflammatory or infected aneurysm. Most patients remain asymptomatic unless the aneurysm has ruptured, causing hypotension and severe acute pain. However, an asymptomatic AAA may be found because of screening, routine physical examination, or imaging studies performed to assess unrelated illnesses. Only 5-22% of AAA cases are reported to be symptomatic; hence, most cases remain asymptomatic until incidentally found [3].

In young patients, the etiology of AAA remains unclear. AAA is closely linked to atherosclerosis and diseases in which arterial wall remodeling and thinning are caused by changes in elastin and collagen and are thought to trigger AAA [4]. According to a study cohort in 2015, 15,411 individuals (aged 45-64 years) who were at risk for AAA had their biomarkers assessed for inflammation, hemostasis, thrombin production, cardiac dysfunction, and vascular stiffness. Results showed 587 cases of AAAs, with smokers (506) accounting for the bulk of cases compared to non-smokers (71) [5]. Smoking can be attributed to the development of AAA, and even though our patient had a brief smoking history from 1999 to 2000, the risk for AAA development, however low, was still seen in people who quit more than a decade ago [6].

Upon extensive research, we were unable to find any cases of asymptomatic AAAs in adults under 40 years of age who lacked underlying risk factors. Most patients with AAA have a high prevalence of cardiovascular risk factors. A study conducted primarily on Indian adults between the ages of 40 and 80 years showed that two out of nine cases that had an incidental diagnosis of AAA were more common in hypertensive men and smokers, and most incidental cases were found to have aortic involvement in the decreasing order of infrarenal (45.4%), juxta-renal (36.4%), and suprarenal (18.1%) [7].

There is considerable uncertainty surrounding these types of cases when screening. Individuals under 65 years of age who present with no symptoms are not subject to a standard protocol, which could result in a general increase in mortality from ruptured AAA in these younger individuals. Patients who report symptoms usually have underlying illnesses, including connective tissue disorders or an infectious etiology linked to AAA. It is important to note that this information is sometimes not properly relayed to referring doctors. Up to one-third of patients with incidentally discovered aneurysms receive no further monitoring and an even lesser number of cases inform their primary care provider that they have an abdominal aneurysm [8].

Once it develops, AAA progression is inevitable. The symptoms are typically minor in the early stages; therefore, a high index of suspicion is required to make a diagnosis. After a diagnosis is made, each patient’s risk of rupture should be assessed against the risk of surgical complications [9]. AAAs dilate at varying rates over time, with the typical aneurysm development rate for minor aneurysms (3.0-5.4 cm) estimated to be 2.21 mm per year [10], and a five-year overall cumulative rupture rate of 25-40% for incidentally diagnosed aneurysms greater than 5.0 cm [11].

Patients with small AAAs measuring 3.0 to 3.9 cm in diameter should undergo duplex ultrasonography for imaging surveillance every three years, while those with aneurysms measuring 4.0 to 4.9 cm in diameter should undergo the procedure once a year, and those with aneurysms measuring 5.0 cm in diameter or larger should undergo the procedure every six months [12]. Smoking cessation is advised to reduce the risk of growth and rupture. Beta-blockers, statins, and other antihypertensive drugs may be recommended to lower cardiovascular risk, but they have not been demonstrated to lower growth and should not be administered for this reason.

Endovascular aortic aneurysm repair (EVAR) remains the mainstay for the treatment of AAAs, as open vascular surgery has been associated with increased 30-day mortality and higher rates of complications in multiple organ systems, including increased duration of stay [13]. Although most follow-up treatments are performed using catheter-based techniques, reintervention rates following EVAR are greater than those observed after open surgical repair [14].

## Conclusions

This case illustrates the possibility of an abdominal aneurysm occurring in asymptomatic young adults with the absence of known major risk factors or underlying disorders. As there is currently no screening protocol for younger individuals to identify AAA, events are only reported when the aneurysm has significantly developed to the point where symptoms occur. The size of an aneurysm is believed to be the largest contributing factor to its rupture. We highlight the importance of ruling out AAA with efficient screening programs, even in younger patients, to timely detect and prevent potentially fatal consequences.
